# Supplementary material for: Topologically controlled synthesis of active colloidal bipeds
Source: Nat Commun. 2024 Jul 9;15:5735. doi: 10.1038/s41467-024-50023-7 (PMC11231253; doi:10.1038/s41467-024-50023-7)
Supplement: Supplementary file 2 — Description of Additional Supplementary Files [file 41467_2024_50023_MOESM2_ESM.pdf]

Supplementary Material for Topologically controlled synthesis of active colloidal bipeds

Jonas Elschner,[1] Farzaneh Farrokhzad,[1] Piotr Kuświk,[2] Maciej Urbaniak,[2] Feliks Stobiecki,[2] Sapida Akhundzada,[3] Arno Ehresmann,[3] Daniel de las Heras,[4] and Thomas M. Fischer[1,*]

[1] *Experimentalphysik X, Physikalisches Institut, Universität Bayreuth, D-95440 Bayreuth, Germany*
[2] *Institute of Molecular Physics, Polish Academy of Sciences, 60-179 Poznań, Poland.*
[3] *Institute of Physics and Center for Interdisciplinary Nanostructure Science and Technology (CINSaT), Universität Kassel, D-34132 Kassel, Germany*
[4] *Theoretische Physik II, Physikalisches Institut, Universität Bayreuth, D-95440 Bayreuth, Germany*

(Dated: June 24, 2024)

This PDF file includes:
Description of Movies S1 to S8
Other Supplementary Materials for this manuscript include the following:
Movies S1 to S8

MOVIE FILES

Supplementary Video 1 Videoclip showing a flyby along action space and control space allowing to see all subloops in control space, including $\mathcal{L}_{\mathrm{CW}}$ which is not visible in figure 1.

Supplementary Video 2 Videoclip showing the response of colloidal particles to the application of the loop $\mathcal{L}_{entry} * \mathcal{L}^2_{\mathrm{e}xit,2}$. The clip shows one biped and four single colloids in the active zone right from the start, the transport of two single colloidal particles toward the active zone (colored in cyan), the growth of one further biped to the outgrown b_2 length and the transport of both outgrown bipeds out of the active zone.

Supplementary Video 3 Videoclip showing the response of colloidal particles to the application of the loop $\mathcal{L}_{entry} * \mathcal{L}_{\mathrm{e}xit,3}$. The clip shows the transport of six single colloidal particles toward the active zone (colored in cyan), the growth of two bipeds to the outgrown b_3 length and the transport of outgrown bipeds out of the active zone.

Supplementary Video 4 Videoclip showing the response of colloidal particles to the application of the loop $\mathcal{L}_{entry} * \mathcal{L}_{\mathrm{e}xit,3\&4} * \mathcal{L}^{-1}_{\mathrm{e}xit,3}$. The clip shows the transport of ten single colloidal particles toward the active zone (colored in cyan), the growth of two bipeds to the outgrown b_4 length, the transport of outgrown bipeds out of the active zone and two single colloids remaining in the active zone.

Supplementary Video 5 Videoclip showing the response of colloidal particles to the application of the loop $\mathcal{L}_{entry} * \mathcal{L}_{\mathrm{e}xit,5}$. The clip shows the transport of eighteen single colloidal particles toward the active zone (colored in cyan), the growth of three bipeds to the outgrown b_5 length, the growth of one biped to a juvenile b_3-biped, and the transport of the three outgrown bipeds out of the active zone.

Supplementary Video 6 Videoclip showing the response of colloidal particles to the application of the loop $\mathcal{L}_{entry} * \mathcal{L}_{\mathrm{e}xit,6}$. The clip shows the transport of several single colloidal particles toward the active zone (colored in cyan), the growth of one biped to the outgrown b_6 length, the transport of the outgrown biped out of the active zone, and the remaining of three single colloids in the active zone.

Supplementary Video 7 Videoclip showing the response of colloidal particles to the application of the loop $\mathcal{L}_{entry} * \mathcal{L}_{\mathrm{e}xit,7}$. The clip shows the transport of twenty single colloidal particles toward the active zone (colored in cyan), the growth of two bipeds to the outgrown b_7 length, the growth of one biped to a juvenile b_6-biped, and the transport of the two outgrown bipeds out of the active zone.

Supplementary Video 8 Videoclip showing a flyby along the action space and control space of figure 4, allowing to see all subloops and all northern excess regions in control space.
